# MIND and Mediterranean Diets Associated with Later Onset of Parkinson's Disease

**DOI:** 10.1002/mds.28464

**Published:** 2021-01-06

**Authors:** Avril Metcalfe‐Roach, Adam C. Yu, Ella Golz, Mihai Cirstea, Kristen Sundvick, Daniel Kliger, Liam H. Foulger, Melissa Mackenzie, B. Brett Finlay, Silke Appel‐Cresswell

**Affiliations:** ^1^ Department of Microbiology and Immunology University of British Columbia Vancouver British Columbia Canada; ^2^ Michael Smith Laboratories UBC Vancouver British Columbia Canada; ^3^ Pacific Parkinson's Research Centre and Djavad Mowafaghian Centre for Brain Health, UBC Vancouver British Columbia Canada; ^4^ Division of Neurology, Faculty of Medicine UBC Vancouver British Columbia Canada; ^5^ Department of Biochemistry and Molecular Biology UBC Vancouver British Columbia Canada

**Keywords:** Parkinson's disease, mediterranean diet, MIND diet, sex differences

## Abstract

**Background:**

The MIND diet has been linked with prevention of Alzheimer's disease and cognitive decline but has not been fully assessed in the context of Parkinson's disease (PD). The objective of the present study was to determine whether MIND diet adherence is associated with the age of Parkinson's disease onset in a manner superior to that of the Mediterranean diet.

**Methods:**

Food Frequency Questionnaires from 167 participants with PD and 119 controls were scored for MIND and 2 versions of Mediterranean diet adherence. Scores were compared between sex and disease subgroups, and PD diet adherence was correlated with age at onset using univariate and multivariate linear models.

**Results:**

The female subgroup adhered more closely to the MIND diet than the male subgroup, and diet scores were not modified by disease status. Later age of onset correlated most strongly with MIND diet adherence in the female subgroup, corresponding to differences of up to 17.4 years (*P* < 0.001) between low and high dietary tertiles. Greek Mediterranean adherence was also significantly associated with later PD onset across all models (*P* = 0.05–0.03). Conversely, only Greek Mediterranean diet adherence remained correlated with later onset across all models in men, with differences of up to 8.4 years (*P* = 0.002).

**Conclusions:**

This cross‐sectional study found a strong correlation between age of onset of PD and dietary habits, suggesting that nutritional strategies may be an effective tool to delay PD onset. Further studies may help to elucidate potential nutrition‐related sex‐specific pathophysiological mechanisms and differential prevalence rates in PD. © 2021 The Authors. *Movement Disorders* published by Wiley Periodicals LLC on behalf of International Parkinson and Movement Disorder Society.

Numerous epidemiological studies have investigated the effects of regional dietary trends on population health and longevity. The Western diet, common in North America, is notorious for its high levels of processed and fried foods, sugar, and red meat; this diet has been linked to increased prevalence and severity of many diseases, including cardiovascular disease (CVD), diabetes, and cancer.^1^, ^2^ Conversely, the Mediterranean diet (MeDi) has garnered significant interest because of its association with reduced rates of cancer,^3^ CVD,^3^ and neurodegenerative diseases such as Alzheimer's disease (AD) and Parkinson's disease (PD).^4^ Two principal MeDi scoring methods exist: the original MeDi (OMeDi) is characterized in part by its antioxidant‐rich mix of vegetables, whole grains, and reduced red meat/dairy^5^ and was revised to promote fish intake, whereas the alternative Greek MeDi (GMeDi) pattern uses similar food groups but also promotes potato intake and limits poultry consumption.^6^

The Mediterranean‐DASH Intervention for Neurodegenerative Delay (MIND) diet, first published in 2015, attempted to refine the MeDi to minimize cognitive decline.^7^ Although the majority of food groups are similar or identical to those found in the MeDi, the MIND diet uniquely rewards leafy green, berry, and poultry intake while minimizing the consumption of fried food and sweets. Milk, potato, and fruit intake are also discarded. The MIND diet has been associated with up to a 54% reduction in AD incidence^7^ and has consistently proven to be more beneficial for cognitive health than the MeDi.^8^, ^9^ Despite this success, little research has investigated the effect of the MIND diet on other neurodegenerative diseases. Agarwal et al (2018) previously showed that higher MIND dietary adherence correlated with reduced incidence and progression of parkinsonian symptoms during aging,^10^ but to date no studies have investigated the potential impact of the diet on patients formally diagnosed with PD. This cross‐sectional study examines the relationship between MIND diet adherence and the age of PD onset in a Canadian cohort and compares the performance of the MIND diet to both MeDi scoring methods.

## 1 Methods

### 1.1 Study Population and Participant Recruitment

Two hundred twenty‐five participants with PD (age of onset within the last 12 years) and 156 control participants were recruited through the Pacific Parkinson's Research Centre (PPRC) at the University of British Columbia (UBC), Canada, using inclusion/exclusion criteria described previously.^11^ Dietary surveys with missing data (n = 93) were not included in the analysis, as well as PD participants with no recorded age of onset (n = 2), leaving a total of 167 PD and 119 control participants. Thirty‐one spousal pairs, all of which consisted of 1 PD and 1 control participant, were identified from the remaining cohort and excluded from all analyses that involved the control group. The study was approved by the UBC Clinical Research Ethics Board and written informed consent was obtained from each participant.

### 1.2 Data Collection

All data were self‐reported and collected either during a study visit or through an online data collection portal. Disease status refers to whether the participant is part of the PD or the control group. Age of onset was defined as the age at which the participant first started to experience motor symptoms as recorded in the chart and supported by self‐report. Dietary patterns over the past year were assessed using the EPIC‐Norfolk Food Frequency Questionnaire (FFQ),^12^ and exercise habits were assessed using the Physical Activity Scale for the Elderly (PASE).^13^ Total energy intake was calculated using the FFQ EPIC Tool for Analysis (FETA)^14^ and is reported in kilocalories (kcal). Smoking habits were categorized as current, previous, and never, and blood pressure was self‐reported as low, normal, or high. History of diabetes (including gestational diabetes) and cardiovascular disease (CVD) were recorded as true or false, as was family history of PD (confirmed cases in first‐degree relatives). PASE and PD family history data were only collected from a subset of the PD cohort (n = 121 and 123, respectively), as it was included after the study had commenced.

### 1.3 Diet Scoring

A list of all food groups and the consumption frequencies used for scoring can be found in the Supplementary data (Tables S1–S3). For all diets, food items that did not fall in any of the listed food groups were discarded. MIND dietary adherence was calculated using the number of servings per food group outlined by Morris et al,^15^ giving MIND scores out of a maximum of 15 for each participant.

For the OMeDi scoring, food groups were binned as specified by Trichopoulou et al,^5^ and the ratio of monounsaturated to saturated fat intake was calculated using FETA.^14^ Participants who consumed below the sex‐specific median for dairy and meat were given a score of 1 for the category or 0 for the remaining categories. Ethanol intake (g/day) was estimated by multiplying the relevant FFQ items by the following ethanol contents: wine, 15 g/glass; beer, 14.4 g/half pint; ports/liqueurs, 10 g/glass; and spirits, 9.2 g/shot. A score of 1 was assigned for consumption between 5 and 25 g/day for women and between 10 and 50 g/day for men. Food group scores were then summed to give the OMeDi score out of a maximum of 9.

For the GMeDi scoring, food groups were scored out of 5 according to Panagiotakos et al.^6^ Ethanol intake (g/day) was quantified as described above and scored out of 5. Accurate quantification of olive oil intake was not available; instead, a score of 3 points was added to the total score if olive oil was the primary cooking oil used by the participant. All categorical scores were then summed to give the GMeDi score out of a maximum of 53.

All dietary tertiles were assigned in a manner that optimized PD participant distribution (Table S4).

### 1.4 Statistical Analysis

All analyses were conducted in R. Univariate associations were queried using Kruskal‐Wallis tests for binary variables and linear regression for continuous variables. All multivariate associations between age of onset (response variable) and dietary score (explanatory variable) were queried using linear regression, whereas associations between sex and dietary score used logistical regression. Dietary score was treated as a continuous variable or with tertiles represented as ordinal factors. Nonparametric differences in the distribution of metadata across tertiles were assessed using Kruskal‐Wallis tests for continuous variables and chi‐square analysis for categorical variables.

## 2 Results

### 2.1 Cohort Statistics

Tables 1 and 2 summarize the overall and tertile‐based descriptive statistics of the PD and control cohorts, respectively. Tables for sex‐specific subgroups can be found in Supplementary Tables S5–S8, along with interaction plots of dietary tertiles with each variable (Fig. S1). Dietary score ranges can be found in Table S9.

**TABLE 1:** PD cohort characteristics

		MIND (/15)				Original MeDi (/9)				Greek MeDi (/53)			
	All	T1	T2	T3	Pval	T1	T2	T3	Pval	T1	T2	T3	Pval
n (total)	167	49	62	56		47	77	43		58	56	53	
Median diet score (IQR)		6 (1)	7.5 (1)	9 (1)		2 (1)	4 (1)	7 (2)		26 (3)	31 (2)	36 (3)	
% Female	31.7	22.4	29	42.9	0.069	31.9	31.2	32.6	0.987	31	32.1	32.1	0.990
Age	64.9	60.9	66.5	66.6	**<0.001**	61.6	66	66.4	**0.003**	62.6	64.6	67.7	**0.002**
Disease duration (years)	6.5	6.5	7	5.8	0.146	6.2	6.5	6.7	0.677	6.4	6.8	6.1	0.408
Age of onset	58.4	54.4	59.5	60.8	**<0.001**	55.4	59.6	59.7	**0.023**	56.2	57.8	61.6	**0.001**
Energy intake (kcal)	1659.7	1466	1748	1731	**0.006**	1566	1605	1860	**0.010**	1645	1632	1706	0.639
Education (years)	16.1	16	16.2	16.2	0.816	15.7	16.4	16.1	0.167	16.1	15.9	16.3	0.392
% Smokers (lifetime)	40	33.3	49.2	35.7	0.177	42.6	44	30.2	0.311	41.4	44.4	34	0.523
Exercise score	161.7	171.5	153.3	162.3	0.466	161.1	145.2	192.5	**0.005**	146.9	182.7	159.4	0.685
% Normal blood pressure	65	63.6	64.2	67.4	0.921	52.5	66.2	76.3	0.465	68	57.4	69.6	0.457
BMI	26.5	28.1	26.4	25.3	**0.004**	27.8	26.7	24.9	**0.006**	27.2	26.5	25.8	0.145
Height	172.8	173.7	173.7	170.8	0.139	173.2	172.5	172.7	0.942	173.5	173.4	171.3	0.464
% Diabetes	6.4	2.9	9.8	5.9	0.482	4.2	11.1	0	0.116	7.9	2.9	8.1	0.607
% CVD	25.7	26.5	30.6	19.6	0.390	38.3	23.4	16.3	**0.047**	32.8	26.8	17	0.161

The all column represents means for the overall cohort, whereas columns T1, T2, and T3 are dietary tertiles. Differences between tertiles were calculated using nonparametric (numerical data) and chi‐square (categorical data) tests.

**TABLE 2:** Control cohort characteristics

		MIND (/15)				Original MeDi (/9)				Greek MeDi (/53)			
	All	T1	T2	T3	Pval	T1	T2	T3	Pval	T1	T2	T3	Pval
n (total)	84	20	30	34		26	31	27		20	38	26	
Median diet score (IQR)		6 (1)	7.5 (1)	9 (1)		2 (1)	5 (1)	6 (1)		26 (4)	31 (2)	37 (4)	
% Female	60.7	35	53.3	82.4	**0.002**	53.8	64.5	63	0.684	50	68.4	57.7	0.366
Age	61.8	62.4	60.1	63.1	0.567	62.6	61.1	62	0.951	66.1	57.8	64.5	**0.003**
Energy intake (kcal)	1569.9	1381	1603	1651	0.092	1372	1650	1668	0.071	1520	1597	1569	0.943
Education (years)	17.1	17.1	16.7	17.4	0.853	16.8	16.6	18.1	0.296	17.6	16.6	17.4	0.432
% Smokers (lifetime)	39.8	47.4	36.7	38.2	0.736	30.8	46.7	40.7	0.476	42.1	52.6	19.2	**0.027**
Exercise score	175.2	186.2	141.2	225.8	0.129	158.2	223.4	167.4	0.862	153.8	168	192	0.645
% Normal Blood pressure	60.4	50	71.4	52.9	0.440	84.6	50	52.9	0.382	57.1	64	56.2	0.905
BMI	26.6	28.4	25.2	27	0.254	26.8	27.5	25.6	0.582	28.2	26.1	26.2	0.209
% Diabetes	9.1	18.2	4.3	9.5	0.421	0	14.3	10	0.349	12.5	10.7	5.3	0.764
% CVD	23.8	30	23.3	20.6	0.733	19.2	25.8	25.9	0.804	10	28.9	26.9	0.247

The all column represents means for the overall cohort, whereas columns T1, T2, and T3 are dietary tertiles. Differences between tertiles were calculated using nonparametric (numerical data) and chi‐square (categorical data) tests.

PD participants were primarily male (68.3%), were an average ± SD of 64.9 ± 8.0 years old, and had begun to experience motor symptoms (referred to as age of onset) an average of 6.5 ± 3.1 years previously. Control participants were only 39.3% male and were slightly younger (mean ± SD, 61.8 ± 9.9 years). PD participants who were older and had later age of onset had higher adherence to all diets; these correlations remained significant only in the MeDi variants for men and the MIND diet for women. In contrast, age was not significantly associated with any dietary score in the corresponding control groups with the exception of the GMeDi, which was nonlinearly associated, driven by women and likely spurious (Wilcox, *P* = 0.003). High OMeDi adherence correlated with lower CVD incidence and higher exercise scores in the sex‐combined PD cohort, whereas high adherence to both the MIND diet and the OMeDi correlated with higher exercise scores in PD women. High MIND diet adherence also corresponded to higher exercise scores in female controls. PD male adherence to all diets correlated with lower body mass index (BMI) values, although OMeDi and MIND diets were also associated with higher kilocalorie consumption in PD men. High GMeDi adherence corresponded with lower smoking rates in the controls overall and lower CVD rates in male controls.

**FIG. 1:**
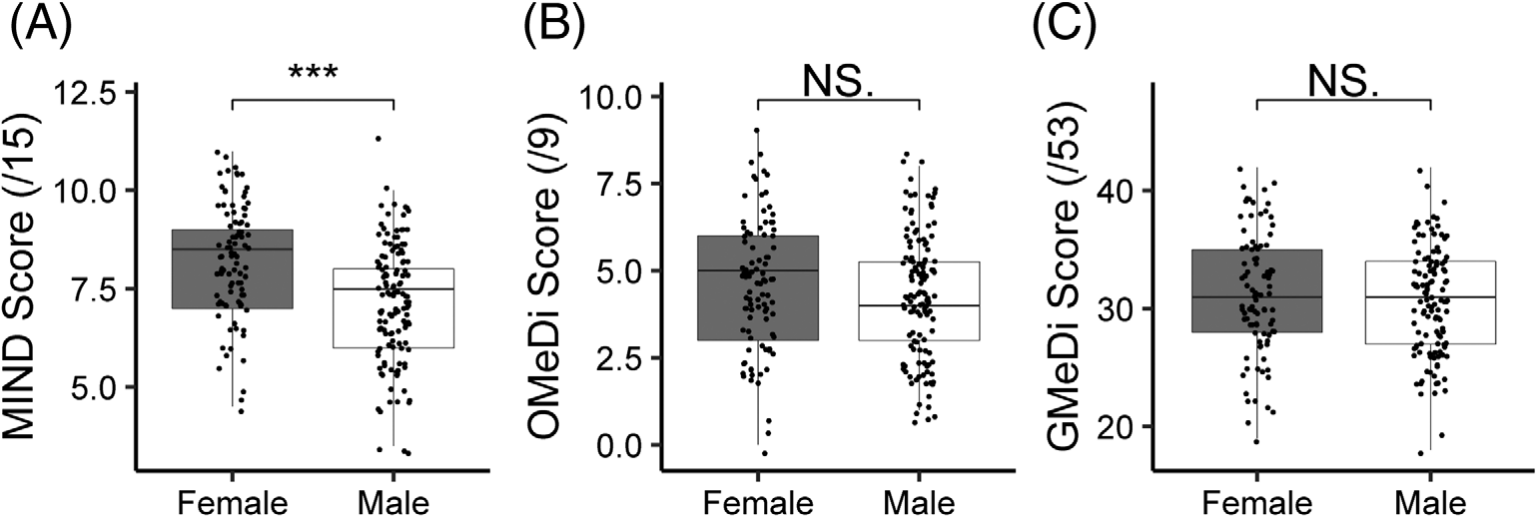
Sex‐stratified scores for (**A**) MIND diet, (**B**) OMeDi, and (**C**) GMeDi. ***P < 0.001. Both PD and control participants are included.

Women scored 1.1 points higher on the MIND diet than men on average (Wilcox, *P* < 0.001), even after controlling for disease status, kilocalories, age, and disease duration (logistic regression, *P* < 0.001). Female PD participants appeared to have slightly lower median MIND scores than their control counterparts and vice versa in the male cohort (Fig. S2); however, these differences were not significant. No other significant associations were observed between other diet scores and sex/disease status (Fig. 1).

### 2.2 MIND Diet Adherence Correlates with Later Disease Onset, Especially Among Women

**FIG. 2:**
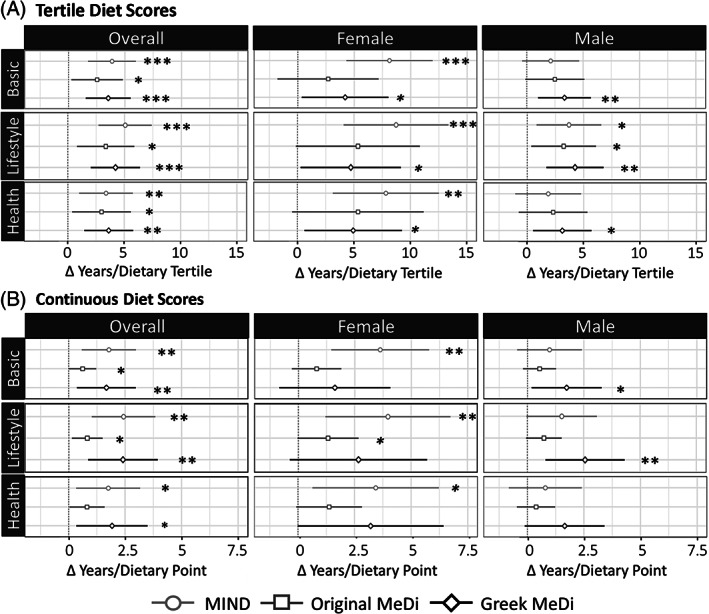
Estimated change in age of PD onset per unit increase in (**A**) tertile and (**B**) continuous (/10) diet scores (β). Multivariate regression was used to calculate all estimates and 95% confidence intervals (represented by lines). *P < 0.05, **P < 0.01, ***P < 0.001.

To facilitate the comparison of model estimates, all dietary scoring systems were adjusted to a 0–10 scale (see Table S9 for score ranges). Three linear regression models were used to query the relationship between dietary adherence and age of onset: basic (n = 167: disease duration, kcal, sex), lifestyle (n = 121: basic + smoking, years of education, exercise), and health (n = 123: basic + high/low blood pressure, diabetes and CVD history, BMI, family PD history). Diet scores were regressed as both continuous adjusted scores (/10) and tertiles; the estimated dietary effects (β) from each model were then compared using effect plots, in which positive estimates indicate a positive correlation between diet adherence and age of onset (Fig. 2). Statistics on all models and corresponding regression plots are included in the Supplementary Table S10 **and** Figure S3.

All results discussed compare the estimated difference in age of onset between the lowest and highest dietary tertiles unless otherwise specified (E = 2*β, presented as the range of model estimates). Although MIND diet adherence correlated most strongly with age of onset in the overall cohort, striking sex‐specific effects were revealed upon stratification. Higher MIND diet adherence correlated far more robustly with later onset in women (E = 15.6–17.4, *P* ≤ 0.003) than in men (E = 3.6–7.4, *P* = 0.21–0.01) or any other diet in either subgroup (E = 4.6–10.8, *P* < 0.25). The GMeDi model in the female subgroup also reached significance, although to a lesser degree (E = 8.4–9.8, *P* = 0.05–0.03). In men, the GMeDi correlated most consistently with age of onset (E = 6.2–8.4, *P* = 0.02–0.002) and was the only diet to remain significantly associated across every model. The MIND diet was only weakly correlated with age of onset (E = 3.6–7.4, *P* = 0.21–0.01), performing similarly to the OMeDi (E = 4.6–6.4, *P* = 0.15–0.03). Similar trends were observed in the tertile and continuous data sets, although OMeDi effect sizes were far smaller because of their wider score range (Table S9).

## 3 Discussion

In this cross‐sectional study, higher adherence to the MIND diet was significantly associated with a higher age at disease onset, especially in women, who had a difference of up to 17.4 years between the highest and lowest tertiles of diet adherence. In men, the GMeDi was consistently more significant than the MIND diet and the OMeDi across models and was associated with up to an 8.4‐year difference in age of onset between low and high tertiles. Although female participants experienced only slightly larger MeDi effect sizes compared with male participants, the average effect size of the MIND diet in women was more than 3 times that of the men and surpassed all MeDi effect sizes, suggesting that its dietary components may be better suited to delaying PD onset than MeDi in a female‐specific manner.

Similarly, only the MIND diet showed any interaction between sex and diet score, despite neither the MIND diet nor the GMeDi normalizing food intake by sex. Female participants adhered significantly closer to the MIND diet than did male participants, even after correcting for age, disease status and duration, and kilocalorie consumption, indicating that the higher MIND score is not simply because of differences in food volume. As the sex difference was similar in the PD and control groups (β = 1.0 and 1.2, respectively), it is unlikely that this effect is an artifact of any sex‐specific dietary shifts that may occur on PD diagnosis. This tendency for women to adhere more strongly to the MIND diet may contribute to their lower rate of PD incidence.

An analysis of 2 large US cohorts found that although GMeDi adherence was only weakly associated with reduced PD risk (*P* = 0.07), the “prudent” dietary pattern was slightly more strongly associated (*P* = 0.04).^16^ Interestingly, this prudent pattern promoted several items such as poultry and leafy vegetables in a manner more similar to the MIND diet than either MeDi. Several other studies have also found negative correlations between PD status or risk and adherence to MeDi‐ or MIND‐type diets.^17^, ^18^ These results are at odds with the present findings, which found no significant interactions between diet and disease status. It is possible that any dietary differences that may have existed between PD and control participants prior to disease onset are corrected on disease diagnosis in a sex‐independent manner; however, the strength of the interactions between age of onset and dietary score suggests that any dietary shifts that may occur on diagnosis do not significantly affect the results.

Apart from age and kilocalorie consumption, the only sex‐specific associations noted between PD dietary score and the model covariables involved exercise in women and BMI in men; thus, the corresponding lifestyle and health models were presumed to be the most accurate predictors of dietary effects in women and men, respectively. Although all 3 models (basic, lifestyle, health) produced similar diet rankings, the health model resulted in slightly lower average effect sizes compared with the lifestyle model in men. It is well documented that the MeDi imparts significant cardiovascular benefits,^1^, ^19^ some of which are sex dependent; for example, improved insulin homeostasis has been observed only in men.^19^ Indeed, significantly reduced CVD incidence was noted in those with high OMeDi scores (Table 1) and trended similarly for the majority of other diet/sex combinations. If MeDi‐type diets delay PD onset in part via their beneficial cardiovascular effects, then controlling for CVD may reduce the apparent effect of the diets, especially in men. Similarly, the higher and more statistically significant effect sizes observed in the lifestyle model in men support the notion that the model covariables are significant disease‐modifying elements. Smoking has long been associated with reduced PD incidence,^20^ and exercise has been shown to change the dopaminergic system in people with Parkinson's disease^21^, ^22^ and reduce motor symptoms.^23^ A growing number of studies, including the large‐scale FINGER study,^24^ have suggested that exercise may be an effective way to reduce neurological decline, especially as part of a combinatorial therapeutic approach.^25^

Although adherence to all diets was strongly associated with lower BMIs in male PD participants, it was also positively associated with higher kilocalorie consumption with no significant changes in exercise habits. As the majority of food groups in each diet reward increased consumption, it is possible that taller people naturally score higher than shorter people while still maintaining similar or lower BMIs because of their higher energy requirements. However, no correlations were found between diet score and height (Table 1). It is likely that people with low dietary scores consume more foods that are not captured by the FFQ, such as prepackaged meals, and thus their kilocalorie consumption is underestimated.

To the best of our knowledge, this is the first study to examine the role of the MIND diet in a strictly PD cohort. Our female PD‐specific findings mirror previous research in AD and cognitive decline, in which the MIND diet has repeatedly proved more effective than MeDi as a preventive measure over several different mixed‐sex study cohorts.^7^, ^9^, ^15^ Interestingly, women represent two‐thirds of all AD cases and may experience more severe cognitive deficits than their male counterparts.^26^ The observed effects of the MIND diet in AD and in women with PD suggest that the diseases share similar sex‐dependent mechanisms that may be modulated by dietary intake. Several previous studies have indicated that certain effects of MeDi are sex specific in neurotypical cohorts, such as inflammation^27^ and reduced CVD risk,^19^ as mentioned previously. In contrast, few studies have previously identified sex‐based differences related to the MIND diet.^28^ Future work will investigate the effects of the MIND diet on other elements of PD etiology including disease progression, inflammatory markers, and gastrointestinal symptoms such as constipation and dysbiosis.

These findings also corroborate a recent longitudinal study by Agarwal et al,^10^ in which participants in the highest MIND dietary tertile developed parkinsonism at a rate 42% below that of the lowest tertile over an average observation period of 4.6 years. This analysis studied the RUSH Memory and Aging Project (MAP) cohort, which was also used to identify a positive correlation between the MIND diet adherence and reduced incidence/progression of cognitive decline^15^ and AD.^7^ Importantly, the MAP cohort is 75% female. Although no sex‐specific effects were reported in these studies, the high proportion of women suggests that the results are more reflective of female physiology. Beyond the sex ratio, the lack of sex‐specific effects observed may be because of several factors. First, the advanced age of the MAP participants (approximately 80, 15 years older than the present cohort) suggests that the sex specificity observed here may be particularly relevant for the delay of neurodegenerative disease in early/mid senium. In addition, parkinsonism is an umbrella term that does not constitute a diagnosis of PD. In the corresponding study, 43% of the participants developed parkinsonism over a mean follow‐up of 4.6 years, which is an order of magnitude higher than the 10‐year PD risk estimate for men aged 75 (2.6%).^29^ It is possible that the more inclusive definition of parkinsonism in the analysis masked any sex‐specific effects that may be particular to PD. Finally, the methods used to detect sex‐specific effects were not specified, and so direct comparisons cannot be made between studies.

Because of the complexity of the diets, the key elements that drive their beneficial effects are poorly understood. It is believed that the power of the diets stems from a complex range of metabolites acting on multiple disease elements; however, significant progress has been made to identify key molecules and metabolites that act on neurodegenerative diseases in reproducible ways. Leafy greens and berries, which are specific to the MIND diet, are rich in antioxidants such as carotenoids, flavonoids, folate, and vitamins C and E, some or all of which have been associated with lower PD/parkinsonism risk and reduced disease progression in both animal models and human cohorts.^30^, ^31^, ^32^, ^33^, ^34^ Conversely, the MeDi diets restrict the intake of all dairy, whereas the MIND diet penalizes only cheese and butter/margarine consumption. Milk consumption has been repeatedly identified as a risk factor for PD, possibly because of increased pesticide exposure; its omission from the MIND diet may contribute to the reduced efficacy of the diet observed in the male cohort. Overall, determining the subtle differences in the metabolic profiles of the different diets may help to unravel elements of PD etiology that are modified by diet in a sex‐specific manner.

Several limitations should be noted with this study. First, all dietary data are cross‐sectional, with only 1 FFQ analyzed per participant. In addition, the analysis assumes that the dietary habits of each participant have not significantly changed over their lifetime. Although there were no differences found between PD and control dietary scores, a prospective study would be required to ensure that all disease‐related dietary fluctuations are accounted for. Second, the berry food group included in the MIND diet is underrepresented by the FFQ, as the only related question assesses strawberries, raspberries, and kiwi fruits and disregards other common berries such as blueberries. Last, there is a strong correlation between the age of the participant and age of onset (*P* < 0.001; Fig. S4), meaning that any interactions between age and dietary score are misattributed to age of onset. This strong interaction is a result of the study design: only patients who had symptoms of PD for 12 years or less were included, resulting in a narrow disease duration range (mean ± SD, 6.5 ± 3.1 years). Despite this limitation, no significant linear correlations were found between age and diet scores in the controls (Table S11), and the results presented here are thus believed to be valid.

We have captured a strong, female‐driven correlation between MIND diet adherence and delayed PD onset in a manner similar or superior to the MeDi. The sex specificities presented here are novel and may prove to be an important contributor to the sex differences observed in PD. Given the findings presented here and in previous articles, individuals should be encouraged to eat a diet rich in fresh vegetables, whole grains, and healthy oils while limiting their intake of dairy, red meat, and sugary/processed foods. These dietary habits should be promoted from an early age, as prodromal features of PD and other neurodegenerative disorders can manifest decades before official diagnosis^35^; in addition, it is currently unknown whether there are critical time windows in which dietary habits are particularly influential on brain health. Overall, these data paint a compelling rationale for interventional and animal‐based studies that investigate the direct impact of Mediterranean‐style diets on PD etiology in a sex‐specific manner. This study should be repeated in a larger, preferably prospective cohort to confirm these findings. Future work will investigate the effect of the diet on other PD symptoms including gut microbial dysbiosis, disease progression, constipation, cognition, and other factors.

## Author Roles

1) Research project: A. Conception, B. Organization, C. Execution;

2) Statistical Analysis: A. Design, B. Execution, C. Review and Critique;

3) Manuscript: A. Writing of the first draft, B. Review and Critique.

A.M.R.: 1A–C, 2A–C, 3A‐B.

A.C.Y., E.G.: 1B–C, 3B.

K.S.: 1A–C.

M.S.C.: 2A,C, 3B.

D.K., L.H.F., M.M.: 1B–C.

B.B.F.: 1A‐C, 2A,C, 3B.

S.A.C.: 1A‐C, 2A,C, 3B.

## Supporting information


**Figure S1.** Interaction plots of diet adherence with all covariables. Tertile and continuous diet scores were used for continuous and categorical variables, respectively. (A, C) PD participant data. (B, D) Control data.

**Figure S2.** Interactions between PD status and diet score in a sex‐specific manner. NS, *P* > 0.05.

**Figure S3.** Tertile distributions of all diet/model combinations. Gray and white indicate female and male data, respectively. Basic, lifestyle, and health contain 167, 121, and 123 PD participants respectively.

**Figure S4.** Interaction between PD participant age and age at disease onset.

**Table S1.** Scoring method for the MIND diet.
**Table S2**. Scoring method for the OMeDi.
**Table S3**. Scoring method for the GMeDi.
**Table S4**. Sample sizes and tertile distributions for all PD models.
**Table S5**. PD female subgroup characteristics.
**Table S6**. PD male subgroup characteristics.
**Table S7**. Control female subgroup characteristics.
**Table S8**. Control male subgroup characteristics.
**Table S9**. Dietary score ranges.
**Table S10**. Statistical summary of all onset‐versus‐diet models.
**Table S11**. Statistical summary of all age‐versus‐diet models.
