# Supplementary figures and images for: MIND and Mediterranean Diets Associated with Later Onset of Parkinson's Disease
Source: Mov Disord. 2021 Jan 6;36(4):977–84. doi: 10.1002/mds.28464 (PMC8248352; doi:10.1002/mds.28464)

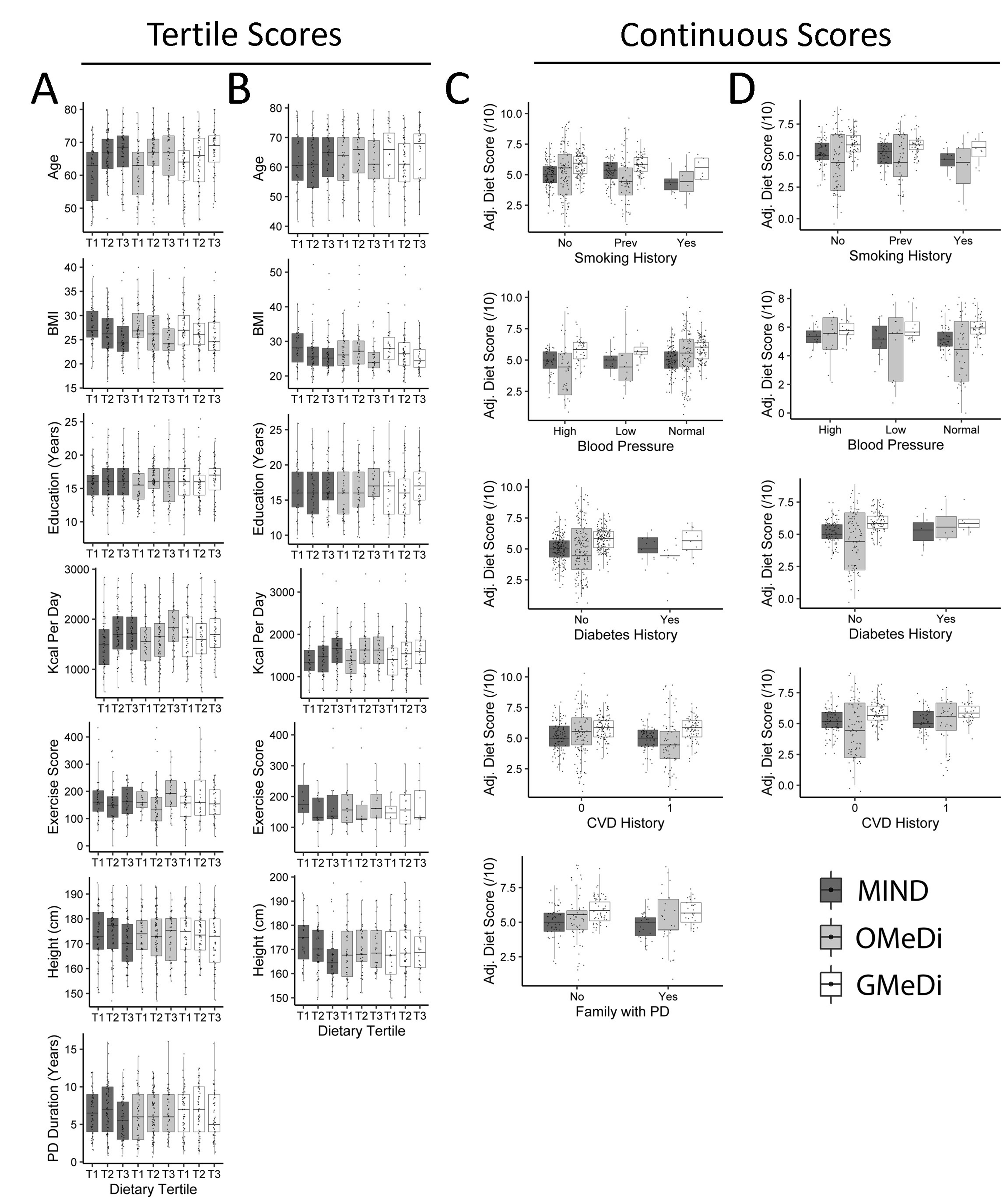

Supplement: Supplementary file 1 — Figure S1. Interaction plots of diet adherence with all covariables. Tertile and continuous diet scores were used for continuous and categorical variables, respectively. (A, C) PD participant data. (B, D) Control data. [file MDS-36-977-s005.tif]

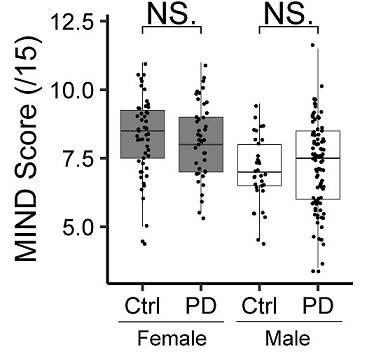

Supplement: Supplementary file 2 — Figure S2. Interactions between PD status and diet score in a sex‐specific manner. NS, P > 0.05. [file MDS-36-977-s001.tif]

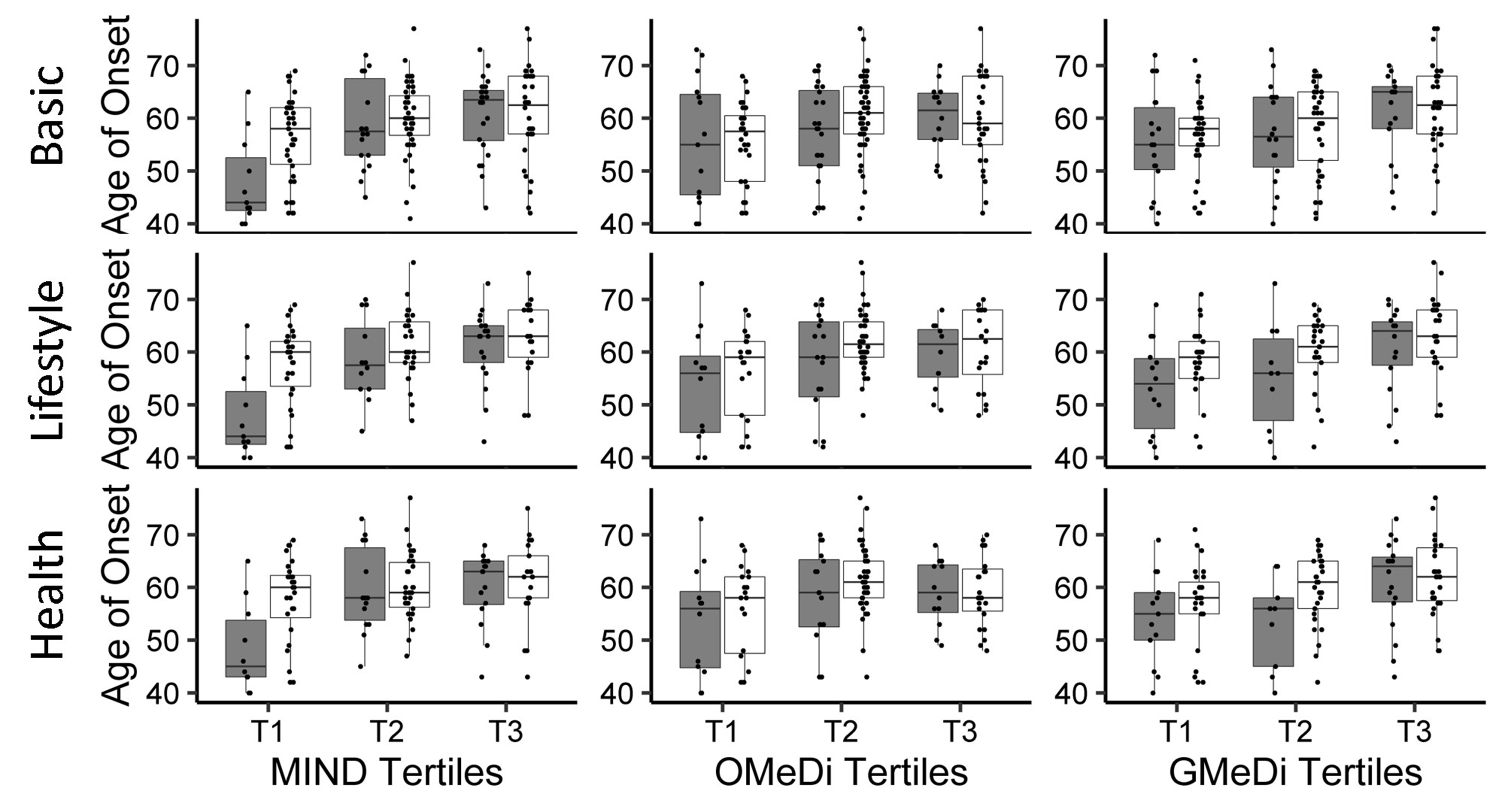

Supplement: Supplementary file 3 — Figure S3. Tertile distributions of all diet/model combinations. Gray and white indicate female and male data, respectively. Basic, lifestyle, and health contain 167, 121, and 123 PD participants respectively. [file MDS-36-977-s002.tif]

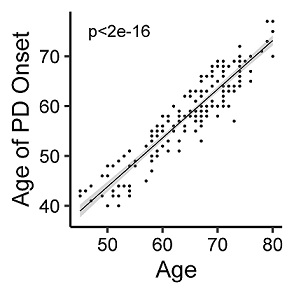

Supplement: Supplementary file 4 — Figure S4. Interaction between PD participant age and age at disease onset. [file MDS-36-977-s004.tif]
