# Supplementary material for: MIND and Mediterranean Diets Associated with Later Onset of Parkinson's Disease
Source: Mov Disord. 2021 Jan 6;36(4):977–84. doi: 10.1002/mds.28464 (PMC8248352; doi:10.1002/mds.28464)
Supplement: Supplementary file 5 — Table S1. Scoring method for the MIND diet. Table S2. Scoring method for the OMeDi. Table S3. Scoring method for the GMeDi. Table S4. Sample sizes and tertile distributions for all PD models. Table S5. PD female subgroup characteristics. Table S6. PD male subgroup characteristics. Table S7. Control female subgroup characteristics. Table S8. Control male subgroup characteristics. Table S9. Dietary score ranges. Table S10. Statistical summary of all onset‐versus‐diet models. Table S11. Statistical summary of all age‐versus‐diet models. [file MDS-36-977-s003.docx]

**Table S1. Scoring method for the MIND diet.**

| MIND | 0 | 0.5 | 1 |
| --- | --- | --- | --- |
| green leafy veg | 0-2 servings/week | 3-5 servings/week | 6+ servings/week |
| other veg | 0-4 servings/week | 5-6 servings/week | 7+ servings/week |
| berries | <1 serving/week | 1-2 servings/week | 3+ servings/week |
| nuts | <1 serving/month | 1/month to 4/week | 5+ servings/week |
| butter & margarine | 3+ tbsp/day | 1-2 tbsp/day | <1 tbsp/day |
| cheese | 7+ servings/week | 1-6 servings/week | <1 serving/week |
| whole grains | <1 serving/day | 1-2 servings/day | 3+ servings/day |
| fish (not fried) | <1 serving/month | 1-3 servings/month | 1+ servings/week |
| beans | <1 serving/week | 1-3 servings/week | 4+ servings/week |
| poultry (not fried) | <1 serving/week | 1 serving/week | 2+ servings/week |
| meat | 7+ servings/week | 4-6 servings/week | <4 servings/week |
| fast/fried food | 4+ times/week | 1-3 times/week | <1 time/week |
| sweets & pastries | 7+ times/week | 5-6 times/week | <5 times/week |
| wine | >1 glass/day or none | 1 glass/month to 6 glasses/week | 1 glass/day |
| olive oil | Not primary cooking oil |  | Primary cooking oil |

**Table S2. Scoring method for the OMeDi.**

| OMeDi | 0 | 1 |
| --- | --- | --- |
| fruits & nuts | Below sex-specific median | At/above sex-specific median |
| Non-starchy vegetables |  |  |
| legumes |  |  |
| cereals & starches |  |  |
| fish |  |  |
| monounsaturated:saturated fat ratio |  |  |
| dairy | At/above sex-specific median | Below sex-specific median |
| meat |  |  |
| alcohol | Any other consumption level | Female: 5-25 g/day  Male: 10-50 g/day |

**Table S3. Scoring method for the GMeDi.**

| GMeDi | 0 | 1 | 2 | 3 | 4 | 5 |
| --- | --- | --- | --- | --- | --- | --- |
| Olive oil | Not primary cooking oil |  |  | Primary cooking oil |  |  |
| alcohol | 0 or 84+ g/day | 72-84 g/day | 60-72 g/day | 48-60 g/day | 36-48 g/day | <36 & >0 g/day |
| fruits | 0/month | 1-4/month | 5-8/month | 9-12/month | 13-18/month | >18/month |
| vegetables (not potatoes) |  |  |  |  |  |  |
| legumes |  |  |  |  |  |  |
| cereals |  |  |  |  |  |  |
| fish |  |  |  |  |  |  |
| potatoes |  |  |  |  |  |  |
| dairy | >18/month | 13-18/month | 9-12/month | 5-8/month | 1-4/month | 0/month |
| meat |  |  |  |  |  |  |
| poultry |  |  |  |  |  |  |

**Table S4. Sample sizes and tertile distributions for all PD models.**

|  |  | Overall | | | Female | | | Male | | |
| --- | --- | --- | --- | --- | --- | --- | --- | --- | --- | --- |
| Tertile | **Diet** | T1 | T2 | T3 | T1 | T2 | T3 | T1 | T2 | T3 |
| Basic (n=167) | MIND | 49 | 62 | 56 | 11 | 18 | 24 | 38 | 44 | 32 |
|  | MeDi (O) | 47 | 77 | 43 | 15 | 24 | 14 | 32 | 53 | 29 |
|  | MeDi (G) | 58 | 56 | 53 | 18 | 18 | 17 | 40 | 38 | 36 |
| Lifestyle (n=121) | MIND | 41 | 42 | 38 | 11 | 12 | 19 | 30 | 30 | 19 |
|  | MeDi (O) | 33 | 56 | 32 | 12 | 18 | 12 | 21 | 38 | 20 |
|  | MeDi (G) | 43 | 35 | 43 | 14 | 10 | 18 | 29 | 25 | 25 |
| CV (n=123) | MIND | 38 | 48 | 37 | 10 | 14 | 16 | 28 | 34 | 21 |
|  | MeDi (O) | 31 | 57 | 35 | 12 | 16 | 12 | 19 | 41 | 23 |
|  | MeDi (G) | 40 | 38 | 45 | 13 | 9 | 18 | 27 | 29 | 27 |

**Table S5. PD female subgroup characteristics.**

|  | | MIND (/15) | | | | Original MeDi (/9) | | | | Greek MeDi (/53) | | | |
| --- | --- | --- | --- | --- | --- | --- | --- | --- | --- | --- | --- | --- | --- |
|  | **All** | **T1** | **T2** | **T3** | **Pval** | **T1** | **T2** | **T3** | **Pval** | **T1** | **T2** | **T3** | **Pval** |
| n (total) | 53 | 11 | 18 | 24 |  | 15 | 24 | 14 |  | 18 | 18 | 17 |  |
| Median Diet Score (IQR) | 8 (2) | 6 (1) | 7.5 (1) | 9 (1) |  | 2 (1) | 5 (1) | 7 (2) |  | 26 (3) | 31 (3) | 36 (2) |  |
| % Female | 100 | 100 | 100 | 100 |  | 100 | 100 | 100 |  | 100 | 100 | 100 |  |
| Age | 63.9 | 54.8 | 65.2 | 67.1 | **<0.001** | 62 | 63.3 | 66.9 | 0.210 | 61.2 | 63.5 | 67.1 | 0.122 |
| Disease Duration (years) | 6.4 | 6.9 | 6.5 | 6.1 | 0.793 | 6.1 | 6.2 | 7 | 0.758 | 6 | 6.7 | 6.6 | 0.809 |
| Age of Onset | 57.5 | 47.9 | 58.7 | 61 | **0.001** | 55.9 | 57 | 59.9 | 0.578 | 55.2 | 56.8 | 60.5 | 0.159 |
| Energy Intake (kcal) | 1634.8 | 1407 | 1670 | 1712 | 0.228 | 1556 | 1583 | 1808 | 0.321 | 1613 | 1684 | 1606 | 0.901 |
| Education (years) | 15.8 | 15 | 15.9 | 16 | 0.628 | 15.3 | 16.5 | 14.9 | 0.054 | 15.6 | 15.4 | 16.3 | 0.324 |
| % Smokers (Lifetime) | 32.1 | 9.1 | 38.9 | 37.5 | 0.185 | 26.7 | 37.5 | 28.6 | 0.739 | 27.8 | 44.4 | 23.5 | 0.370 |
| Exercise Score | 141.8 | 140.3 | 103.5 | 166.4 | **0.021** | 151 | 111.7 | 186.4 | **0.018** | 137.8 | 129.3 | 152.9 | 0.687 |
| % Normal Blood Pressure | 66.7 | 63.6 | 71.4 | 65 | 0.516 | 58.3 | 71.4 | 66.7 | 0.980 | 66.7 | 60 | 73.3 | 0.849 |
| BMI | 25 | 24.6 | 25.7 | 24.7 | 0.950 | 24.5 | 25.8 | 24.2 | 0.838 | 25.2 | 24.8 | 25.1 | 0.972 |
| Height | 162 | 161.5 | 161.5 | 162.6 | 0.794 | 162.8 | 161.4 | 162.1 | 0.929 | 162.3 | 164 | 159.6 | 0.117 |
| % Diabetes | 8.8 | 0 | 10 | 12.5 | 0.589 | 0 | 17.6 | 0 | 0.193 | 9.1 | 0 | 14.3 | 0.499 |
| % Stroke | 8.3 | 0 | 16.7 | 9.1 | 0.552 | 0 | 16.7 | 0 | 0.336 | 11.1 | 0 | 10 | 0.748 |
| % CVD | 22.6 | 27.3 | 27.8 | 16.7 | 0.639 | 40 | 20.8 | 7.1 | 0.103 | 33.3 | 27.8 | 5.9 | 0.124 |
| % Relatives with PD | 12.2 | 20 | 13.3 | 6.2 | 0.573 | 0 | 10 | 27.3 | 0.149 | 0 | 20 | 14.3 | 0.276 |

The All column represents mean values for the female subgroup, whereas columns T1, T2 and T3 are dietary tertiles. Differences between tertiles were calculated using nonparametric (numerical data) and chi-square tests (categorical data).

**Table S6. PD male subgroup characteristics.**

|  | | MIND (/15) | | | | Original MeDi (/9) | | | | Greek MeDi (/53) | | | |
| --- | --- | --- | --- | --- | --- | --- | --- | --- | --- | --- | --- | --- | --- |
|  | **All** | **T1** | **T2** | **T3** | **Pval** | **T1** | **T2** | **T3** | **Pval** | **T1** | **T2** | **T3** | **Pval** |
| n (total) | 114 | 38 | 44 | 32 |  | 32 | 53 | 29 |  | 40 | 38 | 36 |  |
| Median Diet Score (IQR) |  | 6.0 (1) | 7.5 (1) | 9 (1) |  | 2 (1) | 4 (1) | 7 (1) |  | 26 (3) | 31 (2) | 35 (3) |  |
| % Female | 0 | 0 | 0 | 0 |  | 0 | 0 | 0 |  | 0 | 0 | 0 |  |
| Age | 65.4 | 62.7 | 67 | 66.3 | **0.041** | 61.5 | 67.3 | 66.2 | **0.002** | 63.2 | 65.2 | 68 | **0.020** |
| Disease Duration (years) | 6.5 | 6.4 | 7.2 | 5.6 | 0.099 | 6.2 | 6.6 | 6.6 | 0.774 | 6.6 | 6.9 | 5.9 | 0.223 |
| Age of Onset | 58.9 | 56.3 | 59.8 | 60.7 | 0.061 | 55.2 | 60.7 | 59.6 | **0.011** | 56.6 | 58.3 | 62.1 | **0.008** |
| Energy Intake (kcal) | 1671.3 | 1483 | 1780 | 1746 | **0.020** | 1571 | 1615 | 1885 | **0.029** | 1659 | 1607 | 1753 | 0.466 |
| Education (years) | 16.3 | 16.2 | 16.3 | 16.4 | 0.827 | 15.9 | 16.4 | 16.6 | 0.547 | 16.4 | 16.2 | 16.4 | 0.621 |
| % Smokers (Lifetime) | 43.8 | 40.5 | 53.5 | 34.4 | 0.228 | 50 | 47.1 | 31 | 0.267 | 47.5 | 44.4 | 38.9 | 0.748 |
| Exercise Score | 171.2 | 181.4 | 169.8 | 158.4 | 0.857 | 165.3 | 161 | 195.6 | 0.134 | 150.6 | 204.5 | 163.4 | 0.323 |
| % Normal Blood Pressure | 64.3 | 63.6 | 61.5 | 69.2 | 0.383 | 50 | 63.6 | 80.8 | 0.318 | 68.6 | 56.2 | 67.7 | 0.701 |
| BMI | 27.2 | 29.1 | 26.7 | 25.7 | **0.001** | 29.3 | 27 | 25.2 | **<0.001** | 28.1 | 27.3 | 26.1 | **0.035** |
| Height | 177.8 | 177.3 | 178.8 | 177 | 0.372 | 178 | 177.5 | 177.9 | 0.864 | 178.6 | 177.8 | 176.8 | 0.543 |
| % Diabetes | 5.3 | 3.8 | 9.7 | 0 | 0.319 | 5.9 | 8.1 | 0 | 0.415 | 7.4 | 4 | 4.3 | 0.834 |
| % Stroke | 5.6 | 0 | 12 | 0 | 0.158 | 0 | 3.2 | 14.3 | 0.236 | 0 | 6.2 | 10.5 | 0.363 |
| % CVD | 27.2 | 26.3 | 31.8 | 21.9 | 0.623 | 37.5 | 24.5 | 20.7 | 0.283 | 32.5 | 26.3 | 22.2 | 0.597 |
| % Relatives with PD | 14 | 16.7 | 11.4 | 14.3 | 0.830 | 17.4 | 12.2 | 13.6 | 0.846 | 16.7 | 3.7 | 20.7 | 0.162 |

The All column represents mean values for the male subgroup, whereas columns T1, T2 and T3 are dietary tertiles. Differences between tertiles were calculated using nonparametric (numerical data) and chi-square tests (categorical data).

**Table S7. Control female subgroup characteristics.**

|  | | MIND (/15) | | | | Original MeDi (/9) | | | | Greek MeDi (/53) | | | |
| --- | --- | --- | --- | --- | --- | --- | --- | --- | --- | --- | --- | --- | --- |
|  | **All** | **T1** | **T2** | **T3** | **Pval** | **T1** | **T2** | **T3** | **Pval** | **T1** | **T2** | **T3** | **Pval** |
| n (total) | 51 | 7 | 16 | 28 |  | 14 | 20 | 17 |  | 10 | 26 | 15 |  |
| Median Diet Score (IQR) |  | 6 (2) | 7.5 (1) | 9 (1) |  | 2 (1) | 5 (1) | 6 (1) |  | 25 (5) | 31 (2) | 39 (4) |  |
| % Female | 100 | 100 | 100 | 100 |  | 100 | 100 | 100 |  | 100 | 100 | 100 |  |
| Age | 61.3 | 58 | 60.1 | 62.7 | 0.424 | 60.6 | 61.5 | 61.7 | 0.870 | 64.8 | 57.5 | 65.7 | **0.012** |
| Energy Intake (kcal) | 1591.1 | 1419 | 1500 | 1686 | 0.190 | 1439 | 1529 | 1789 | 0.084 | 1585 | 1564 | 1642 | 0.830 |
| Education (years) | 17.2 | 16.3 | 16.7 | 17.7 | 0.534 | 16.4 | 16.6 | 18.4 | 0.240 | 17.5 | 16.9 | 17.5 | 0.847 |
| % Smokers (Lifetime) | 38 | 50 | 31.2 | 39.3 | 0.706 | 35.7 | 36.8 | 41.2 | 0.944 | 44.4 | 46.2 | 20 | 0.228 |
| Exercise Score | 176.4 | - | 128.3 | 240.6 | **0.028** | 146.7 | 247.5 | 149.2 | 0.793 | 123.3 | 170.6 | 205.8 | 0.440 |
| % Normal Blood Pressure | 54.8 | 66.7 | 61.5 | 46.7 | 0.349 | 85.7 | 42.9 | 50 | 0.375 | 60 | 62.5 | 40 | 0.419 |
| BMI | 26 | 25.7 | 24.2 | 27 | 0.525 | 26 | 26.8 | 25 | 0.631 | 26.1 | 25.9 | 25.9 | 0.482 |
| % Diabetes | 2.9 | 0 | 0 | 5.6 | 0.633 | 0 | 7.1 | 0 | 0.479 | 0 | 5.6 | 0 | 0.633 |
| % CVD | 23.5 | 14.3 | 25 | 25 | 0.825 | 7.1 | 35 | 23.5 | 0.169 | 10 | 19.2 | 40 | 0.170 |

The All column represents mean values for the overall cohort, whereas columns T1, T2 and T3 are dietary tertiles. Differences between tertiles were calculated using nonparametric (numerical data) and chi-square tests (categorical data).

**Table S8. Control male subgroup characteristics.**

|  | | MIND (/15) | | | | Original MeDi (/9) | | | | Greek MeDi (/53) | | | |
| --- | --- | --- | --- | --- | --- | --- | --- | --- | --- | --- | --- | --- | --- |
|  | **All** | **T1** | **T2** | **T3** | **Pval** | **T1** | **T2** | **T3** | **Pval** | **T1** | **T2** | **T3** | **Pval** |
| n (total) | 33 | 13 | 14 | 6 |  | 12 | 11 | 10 |  | 10 | 12 | 11 |  |
| Median Diet Score (IQR) |  | 6.5 (1) | 7.5 (0.5) | 8.5 (0.5) |  | 2 (1) | 5 (1) | 6 (0.0) |  | 27 (1) | 31 (2) | 36 (2.5) |  |
| % Female | 0 | 0 | 0 | 0 |  | 0 | 0 | 0 |  | 0 | 0 | 0 |  |
| Age | 62.6 | 64.5 | 60 | 64.8 | 0.638 | 64.9 | 60.4 | 62.4 | 0.744 | 67.3 | 58.5 | 62.9 | 0.143 |
| Energy Intake (kcal) | 1537.2 | 1361 | 1721 | 1490 | 0.554 | 1294 | 1871 | 1461 | 0.109 | 1454 | 1668 | 1470 | 0.925 |
| Education (years) | 17 | 17.6 | 16.8 | 16.2 | 0.651 | 17.3 | 16.5 | 17.4 | 0.957 | 17.8 | 15.8 | 17.4 | 0.487 |
| % Smokers (Lifetime) | 42.4 | 46.2 | 42.9 | 33.3 | 0.870 | 25 | 63.6 | 40 | 0.170 | 40 | 66.7 | 18.2 | 0.062 |
| Exercise Score | 173.5 | 186.2 | 166.9 | 136.9 | 0.984 | 169.7 | 126.9 | 190 | 0.625 | 214.7 | 164.3 | 174.8 | 0.446 |
| % Normal Blood Pressure | 70.6 | 42.9 | 87.5 | 100 | 0.302 | 83.3 | 75 | 57.1 | 0.250 | 50 | 66.7 | 83.3 | 0.083 |
| BMI | 27.7 | 29.6 | 26.3 | 26.8 | 0.419 | 27.7 | 28.6 | 26.6 | 0.552 | 30.1 | 26.5 | 26.7 | 0.329 |
| % Diabetes | 19 | 25 | 10 | 33.3 | 0.574 | 0 | 28.6 | 25 | 0.367 | 33.3 | 20 | 12.5 | 0.731 |
| % CVD | 24.2 | 38.5 | 21.4 | 0 | 0.182 | 33.3 | 9.1 | 30 | 0.351 | 10 | 50 | 9.1 | **0.033** |

The All column represents mean values for the overall cohort, whereas columns T1, T2 and T3 are dietary tertiles. Differences between tertiles were calculated using nonparametric (numerical data) and chi-square tests (categorical data).

**Table S9. Dietary Score Ranges.**

| Status | Diet | Range | | | Adj. Range (/10) | | |
| --- | --- | --- | --- | --- | --- | --- | --- |
|  |  | Overall | Female | Male | Overall | Female | Male |
| PD | MIND (/15) | 3.5-11.5 | 5-11 | 3.5-11.5 | 2.3-7.7 | 3.0-7.3 | 2.3-7.7 |
|  | Original MeDi (/9) | 1-9 | 1-9 | 1-8 | 1.1-10.0 | 1.1-10.0 | 1.1-8.9 |
|  | Greek MeDi (/53) | 18-45 | 19-42 | 18-45 | 3.4-8.5 | 3.6-7.9 | 3.4-8.5 |
| Ctrl | MIND (/15) | 4.5-11 | 4.5-11 | 4.5-9.5 | 3-7.3 | 3-7.3 | 3-6.3 |
|  | Original MeDi (/9) | 0-8 | 0-8 | 1-7 | 0-8.9 | 0-8.9 | 1.1-7.8 |
|  | Greek MeDi (/53) | 20-42 | 20-41 | 23-42 | 3.8-7.9 | 3.8-7.7 | 4.3-7.9 |

Maximum dietary scores are listed under Diet. Adjusted range normalizes all scores to a maximum to 10 to facilitate comparison.

| **Table S10. Statistical summary of all onset vs. diet models.**   \|  \|  \|  \| Overall \| \| \| Female \| \| \| Male \| \| \| \| --- \| --- \| --- \| --- \| --- \| --- \| --- \| --- \| --- \| --- \| --- \| --- \| \| Model \| **Type** \| **Diet** \| **Pval** \| **Estimate (β)** \| **E (2*β)** \| **Pval** \| **Estimate (β)** \| **E (2*β)** \| **Pval** \| **Estimate (β)** \| **E (2*β)** \| \| Basic \| Tert \| MIND \| <0.001 \| 3.8 \| 7.6 \| <0.001 \| 8.1 \| 16.2 \| 0.105 \| 2.1 \| 4.2 \| \| Basic \| Tert \| OMeDi \| 0.031 \| 2.5 \| 5 \| 0.246 \| 2.7 \| 5.4 \| 0.065 \| 2.5 \| 5 \| \| Basic \| Tert \| GMeDi \| <0.001 \| 3.5 \| 7 \| 0.038 \| 4.2 \| 8.4 \| 0.006 \| 3.3 \| 6.6 \| \| Lifestyle \| Tert \| MIND \| <0.001 \| 5.1 \| 10.2 \| <0.001 \| 8.7 \| 17.4 \| 0.014 \| 3.7 \| 7.4 \| \| Lifestyle \| Tert \| OMeDi \| 0.011 \| 3.3 \| 6.6 \| 0.065 \| 5.3 \| 10.6 \| 0.031 \| 3.2 \| 6.4 \| \| Lifestyle \| Tert \| GMeDi \| <0.001 \| 4.2 \| 8.4 \| 0.046 \| 4.7 \| 9.4 \| 0.002 \| 4.2 \| 8.4 \| \| CV \| Tert \| MIND \| 0.006 \| 3.4 \| 6.8 \| 0.003 \| 7.8 \| 15.6 \| 0.219 \| 1.8 \| 3.6 \| \| CV \| Tert \| OMeDi \| 0.027 \| 3 \| 6 \| 0.081 \| 5.4 \| 10.8 \| 0.145 \| 2.3 \| 4.6 \| \| CV \| Tert \| GMeDi \| 0.001 \| 3.6 \| 7.2 \| 0.032 \| 4.9 \| 9.8 \| 0.022 \| 3.1 \| 6.2 \| \| Basic \| Cont \| MIND \| 0.005 \| 1.7 \| - \| 0.002 \| 3.7 \| - \| 0.215 \| 0.9 \| - \| \| Basic \| Cont \| OMeDi \| 0.033 \| 0.6 \| - \| 0.106 \| 0.9 \| - \| 0.150 \| 0.5 \| - \| \| Basic \| Cont \| GMeDi \| 0.009 \| 1.7 \| - \| 0.169 \| 1.7 \| - \| 0.026 \| 1.7 \| - \| \| Lifestyle \| Cont \| MIND \| 0.002 \| 2.2 \| - \| 0.008 \| 4 \| - \| 0.060 \| 1.5 \| - \| \| Lifestyle \| Cont \| OMeDi \| 0.012 \| 0.9 \| - \| 0.039 \| 1.4 \| - \| 0.074 \| 0.7 \| - \| \| Lifestyle \| Cont \| GMeDi \| 0.002 \| 2.5 \| - \| 0.077 \| 2.8 \| - \| 0.006 \| 2.6 \| - \| \| CV \| Cont \| MIND \| 0.023 \| 1.6 \| - \| 0.022 \| 3.4 \| - \| 0.338 \| 0.8 \| - \| \| CV \| Cont \| OMeDi \| 0.052 \| 0.7 \| - \| 0.073 \| 1.4 \| - \| 0.380 \| 0.4 \| - \| \| CV \| Cont \| GMeDi \| 0.012 \| 2 \| - \| 0.060 \| 3.2 \| - \| 0.072 \| 1.6 \| - \| |
| --- | --- | --- | --- | --- | --- | --- | --- | --- | --- | --- | --- | --- | --- | --- | --- | --- | --- | --- | --- | --- | --- | --- | --- | --- | --- | --- | --- | --- | --- | --- | --- | --- | --- | --- | --- | --- | --- | --- | --- | --- | --- | --- | --- | --- | --- | --- | --- | --- | --- | --- | --- | --- | --- | --- | --- | --- | --- | --- | --- | --- | --- | --- | --- | --- | --- | --- | --- | --- | --- | --- | --- | --- | --- | --- | --- | --- | --- | --- | --- | --- | --- | --- | --- | --- | --- | --- | --- | --- | --- | --- | --- | --- | --- | --- | --- | --- | --- | --- | --- | --- | --- | --- | --- | --- | --- | --- | --- | --- | --- | --- | --- | --- | --- | --- | --- | --- | --- | --- | --- | --- | --- | --- | --- | --- | --- | --- | --- | --- | --- | --- | --- | --- | --- | --- | --- | --- | --- | --- | --- | --- | --- | --- | --- | --- | --- | --- | --- | --- | --- | --- | --- | --- | --- | --- | --- | --- | --- | --- | --- | --- | --- | --- | --- | --- | --- | --- | --- | --- | --- | --- | --- | --- | --- | --- | --- | --- | --- | --- | --- | --- | --- | --- | --- | --- | --- | --- | --- | --- | --- | --- | --- | --- | --- | --- | --- | --- | --- | --- | --- | --- | --- | --- | --- | --- | --- | --- | --- | --- | --- | --- | --- | --- | --- | --- | --- | --- | --- | --- | --- | --- | --- | --- | --- | --- | --- | --- | --- | --- | --- | --- | --- | --- | --- | --- | --- | --- | --- | --- | --- | --- |
|  |

| Tert and Cont represent dietary tertiles and continuous dietary scores (/10) respectively. Estimate represents the change in age of onset with each diet point/tertile increase. E represents the estimated difference in age of onset between the lowest and highest tertile. |
| --- |

| **Table S11. Statistical summary of all age vs. diet models.**   \| Model \| Diet \| Sex \| Pval (PD) \| Pval (Ctrl) \| β (PD) \| β (Ctrl) \| \| --- \| --- \| --- \| --- \| --- \| --- \| --- \| \| Tertiles \| MIND \| Overall \| **0.009** \| 0.568 \| 3.1 \| 1.3 \| \| Tertiles \| MIND \| Female \| **0.001** \| 0.311 \| 8.8 \| 3.1 \| \| Tertiles \| MIND \| Male \| 0.220 \| 0.923 \| 1.7 \| 0.4 \| \| Tertiles \| OMeDi \| Overall \| **0.016** \| 0.855 \| 3.1 \| -0.4 \| \| Tertiles \| OMeDi \| Female \| 0.384 \| 0.883 \| 2.3 \| 0.4 \| \| Tertiles \| OMeDi \| Male \| **0.020** \| 0.621 \| 3.4 \| -1.7 \| \| Tertiles \| GMeDi \| Overall \| **0.003** \| 0.584 \| 3.3 \| -1.1 \| \| Tertiles \| GMeDi \| Female \| 0.162 \| 0.796 \| 3 \| 0.7 \| \| Tertiles \| GMeDi \| Male \| **0.007** \| 0.345 \| 3.6 \| -3.1 \| \| Cont (/10) \| MIND \| Overall \| **0.038** \| 0.225 \| 1.3 \| 1.5 \| \| Cont (/10) \| MIND \| Female \| **0.037** \| 0.202 \| 2.7 \| 1.8 \| \| Cont (/10) \| MIND \| Male \| 0.243 \| 0.837 \| 0.8 \| 0.5 \| \| Cont (/10) \| OMeDi \| Overall \| **0.032** \| 0.844 \| 0.7 \| 0.1 \| \| Cont (/10) \| OMeDi \| Female \| 0.263 \| 0.620 \| 0.7 \| 0.3 \| \| Cont (/10) \| OMeDi \| Male \| **0.061** \| 0.713 \| 0.7 \| -0.4 \| \| Cont (/10) \| GMeDi \| Overall \| **0.007** \| 0.948 \| 1.9 \| 0.1 \| \| Cont (/10) \| GMeDi \| Female \| 0.317 \| 0.430 \| 1.3 \| 1.1 \| \| Cont (/10) \| GMeDi \| Male \| **0.008** \| 0.361 \| 2.2 \| -2 \| |
| --- | --- | --- | --- | --- | --- | --- | --- | --- | --- | --- | --- | --- | --- | --- | --- | --- | --- | --- | --- | --- | --- | --- | --- | --- | --- | --- | --- | --- | --- | --- | --- | --- | --- | --- | --- | --- | --- | --- | --- | --- | --- | --- | --- | --- | --- | --- | --- | --- | --- | --- | --- | --- | --- | --- | --- | --- | --- | --- | --- | --- | --- | --- | --- | --- | --- | --- | --- | --- | --- | --- | --- | --- | --- | --- | --- | --- | --- | --- | --- | --- | --- | --- | --- | --- | --- | --- | --- | --- | --- | --- | --- | --- | --- | --- | --- | --- | --- | --- | --- | --- | --- | --- | --- | --- | --- | --- | --- | --- | --- | --- | --- | --- | --- | --- | --- | --- | --- | --- | --- | --- | --- | --- | --- | --- | --- | --- | --- | --- | --- | --- | --- | --- | --- |
|  |

Models included kcal, sex, and disease duration where applicable. Tertiles and Cont refer to dietary tertiles and continuous dietary scores (/10) respectively. Their interaction with age is described using β and p values for PD and control cohorts as specified.
